# Surgical repair of a depressed, comminuted fracture of the zygomatic process of the frontal bone using a locking compression plate in a Thoroughbred colt foal

**DOI:** 10.1002/ccr3.1923

**Published:** 2018-11-20

**Authors:** Ann M. Derham, Jessica P. Johnson, Clodagh M. Kearney, John M. O’Leary

**Affiliations:** ^1^ University College Dublin, University Veterinary Hospital, UCD Dublin 4 Ireland; ^2^ Equine Veterinary Medical Centre Qatar Foundation Doha Qatar

**Keywords:** horse, locking compression plate, palpebral reflex, periorbital fracture

## Abstract

This case report demonstrates the use of a 10‐hole 2.7‐mm locking compression plate (LCP) to repair a depressed, comminuted fracture of the zygomatic process of the frontal bone, in a foal. LCP fixation resulted in excellent cosmesis. The use of LCP fixation in this region has not been previously described.

## 1 INTRODUCTION

This case report demonstrates the use of a 10‐hole 2.7‐mm locking compression plate (LCP) to repair a depressed, comminuted fracture of the zygomatic process of the frontal bone, in a 4‐month‐old Thoroughbred colt foal. Loss of motor function of the right upper eyelid was noted following placement of the LCP and persisted for 5 weeks, the LCP was removed, and intensive medical management of the eye and two temporary tarsorrhaphies were performed over the course of 10 weeks, while motor function very slowly, but gradually improved. The LCP fixation resulted in an excellent cosmetic outcome. Eyelid motor function was almost completely restored 15 weeks after initial trauma to the zygomatic process, avoiding the need for a permanent lateral tarsorrhaphy. This case report demonstrates the excellent cosmetic outcome and healing of a depressed, comminuted fracture in this area. It also suggests that intensive medical management and a prolonged rehabilitation period may be beneficial in cases where cosmetic outcome is a significant factor.

Periorbital fractures are common in horses and often associated with trauma. Most minimally displaced fractures of the zygomatic process of the frontal bone heal without fixation. However, displaced fractures may result in ocular injury (corneal ulceration, exophthalmos, globe rupture, blindness etc), nonhealing wounds due to instability or sequestration/infection, and/or poor cosmetic outcome. Surgical reduction and stabilization is often recommended for such cases.^1^, ^2^, ^3^, ^4^, ^5^, ^6^, ^7^, ^8^, ^9^ To the authors’ knowledge, there is no report documenting the use of an LCP in such a location.

## 2 HISTORY, CLINICAL FINDINGS, AND DIAGNOSIS

A 4‐month‐old, Thoroughbred colt presented with moderate right periorbital swelling and exophthalmos of 1 week's duration. The foal was managed medically with no improvement in swelling noted. The referring veterinarian suspected a right zygomatic arch fracture on palpation and ultrasonographic examination. The foal was referred for surgical repair.

**Figure 1:**
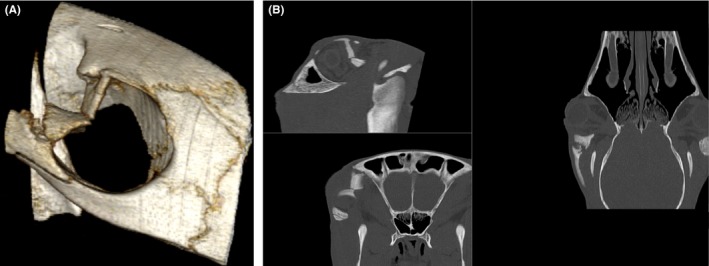
A, CT 3‐D reconstruction of the depressed comminuted fracture of the right zygomatic process of the frontal bone. B, CT MPR of fracture in standard plane, the images are entitled with the WW (window width) and WL (window level). Top left = sagittal plane,bottom left = transverse plane, right = dorsal plane

Upon admittance to the hospital, a depression over the right orbital rim was palpable. Moderate soft tissue swelling was present. Ophthalmic examination, including fluorescein dye staining of the cornea, revealed no ocular lesions, and vision appeared intact. The degree of periorbital swelling made assessment of upper eyelid tone difficult, and complete assessment of functional menace response and palpebral reflex was not possible. A computed tomography (CT) examination was conducted to aid surgical planning and confirmed a depressed, comminuted fracture of the right zygomatic process of the frontal bone with separation of the suture between the zygomatic process of the frontal bone and zygomatic process of the temporal bone (Figure 1A,B).

## 3 TREATMENT

**Figure 2:**
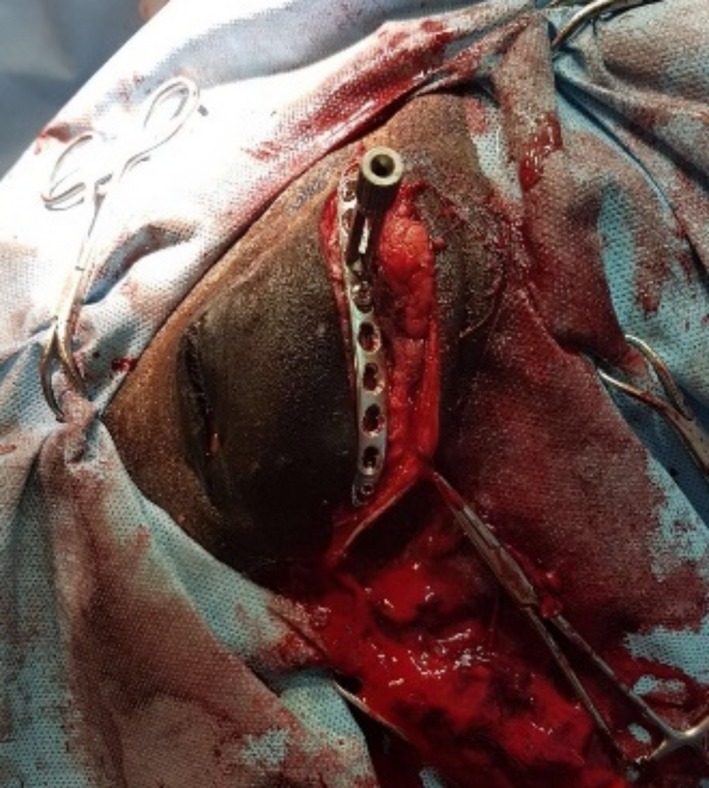
Intraoperative image demonstrating placement of the 2.7‐mm LCP

The foal received a 5‐day perioperative course of ceftiofur 5 mg/kg intravenously (IV) (Wondercef, FATRO, Italy), gentamicin 6.6 mg/kg IV (Gentaject 10%, Franklin Pharmaceuticals, Ireland), flunixin meglumine 1.1 mg/kg IV (Flunixin injection, Norbrook, Ireland), omeprazole 4.4 mg/kg orally (Peptizole, Norbrook), topical ophthalmic chloromycetin (Chloramphenicol 0.5% Eye drops solution, Mercury Pharmaceuticals, UK), and artificial tears (Vidisic, Bausch and Lomb, UK). The foal was anesthetized routinely and placed in left lateral recumbency. A curvilinear skin incision was made over the zygomatic arch, and sharp dissection performed to expose the fracture site. Due to the degree of swelling in the area, we were unable to palpate the auriculopalpebral branch of the palpebral nerve. The fracture was repaired using a precontoured 10‐hole 2.7‐mm LCP (Figure 2). In total, six locking head screws and two cortical screws were placed. Cortex screws were placed in neutral as there was no fracture gap once the fracture was manually reduced. Screw length was full thickness of the zygomatic process of the frontal bone. Digital palpation of the underside of the zygomatic process was performed to ensure no screw was impinging on the globe. No screws were placed in the two holes over the fracture lines. The incision was closed in three layers in a simple continuous pattern, with intradermal skin sutures (USP 2‐0 polydioxanone, Johnson and Johnson, Belgium). Sterile saline lavage of the globe was performed throughout the procedure. Hand‐assisted recovery from anesthesia was uneventful.

## 4 CASE PROGRESSION

During the first 5 days postoperatively, periorbital swelling gradually resolved; however, a significant deficit in motor function of the right upper eyelid with a negative palpebral reflex was diagnosed, which improved only slightly over this period. Fluorescein staining revealed a central superficial corneal ulcer. Topical eye treatments were increased to every 4 hours for a further 10 days. Anti‐inflammatories were switched to meloxicam paste 0.6 mg/kg PO (Loxicom, Norbrook).

### 4.1 Five weeks postoperatively

**Figure 3:**
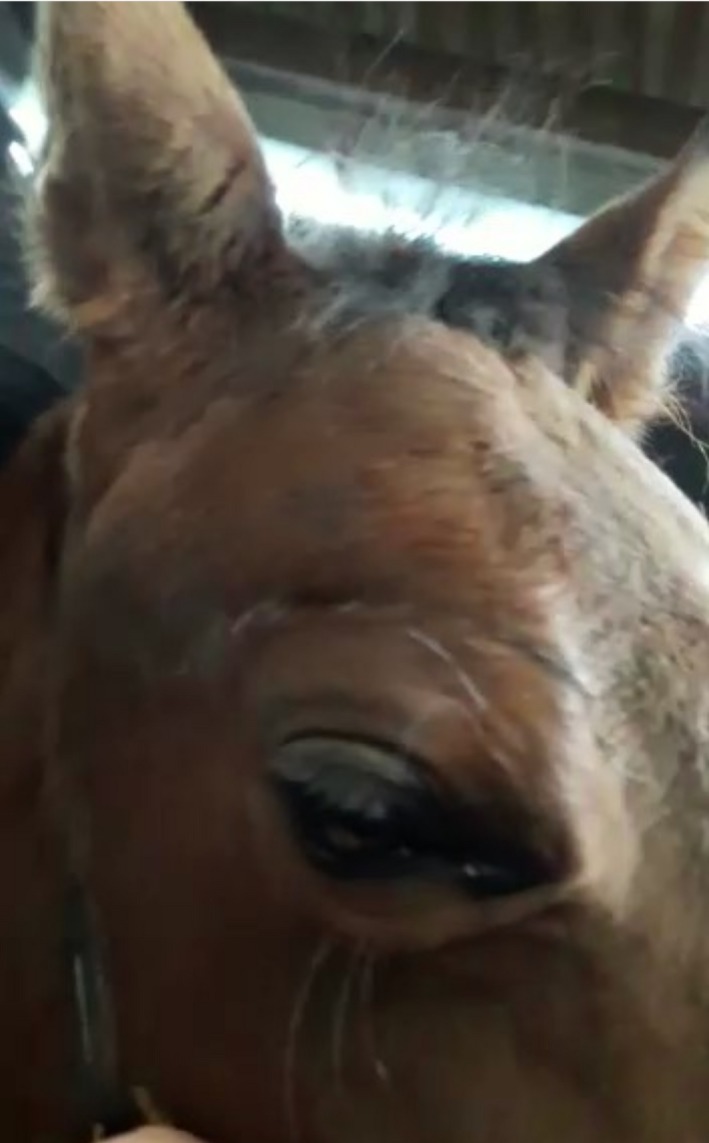
Postoperative image showing excellent cosmesis following repair

Radiographs indicated adequate healing of the fracture, with excellent cosmesis (Figure 3). However, there was still no palpebral reflex present. A presumed diagnosis of iatrogenic damage to the palpebral branch of the auriculopalpebral branch of the facial nerve was made. The foal was anesthetized; the LCP was removed, and a right‐sided temporary lateral tarsorrhaphy was performed. Despite assisted anesthetic recovery, the foal fell during this, resulting in a Type 1b olecranon fracture,^10^, ^11^ this was repaired the following day using a narrow 4.5‐mm 10‐hole dynamic compression plate (DCP). The foal was ambulating comfortably following anesthetic recovery.

### 4.2 Seven weeks postoperatively

The temporary lateral tarsorrhaphy was removed. The palpebral reflex had improved slightly. Artificial tears were continued for a further 2 weeks.

### 4.3 Nine weeks postoperatively

Blepharospasm and epiphora were present in the right eye. Fluorescein staining revealed a large central superficial corneal ulcer. A second temporary lateral tarsorrhaphy was performed under standing sedation and local anesthesia.

### 4.4 Thirteen weeks postoperatively

Follow‐up radiographs indicated good healing of the olecranon fracture, and walking exercise commenced. Upper eyelid motor function was present, with associated loosening of the temporary tarsorrhaphy sutures. A permanent tarsorrhaphy was schedule to be performed in 4 weeks’ time, in conjunction with removal of the elbow plate. The eye was managed medically in the interim period with topical chloromycetin and artificial tears.

## 5 OUTCOME

### 5.1 Fifteen weeks postoperatively

Upper eyelid function was almost completely restored to the upper right eyelid, with a normal palpebral reflex now present. Unfortunately, the foal developed severe juvenile osteoarthritic changes of the proximal interphalangeal joint in the contralateral limb to the olecranon fracture and was euthanized.

## 6 DISCUSSION

Orbital fractures can threaten the globe's integrity and result in obvious cosmetic defects. Conservative management of this type of fracture would have resulted in a poor cosmetic outcome and potential exposure keratopathy.^1^, ^2^, ^3^, ^4^, ^5^, ^6^, ^7^, ^8^, ^9^, ^12^ Several surgical stabilization techniques have been reported for this fracture configuration. Tension‐band wiring is frequently used in periorbital fractures,^2^, ^5^, ^7^ but is unlikely to provide adequate support for comminuted fractures. Three previous reports have demonstrated successful cosmetic outcomes of periorbital fractures following plate fixation, using a cuttable bone plate, T‐plate, and resorbable plate and tissue expanders.^5^, ^13^, ^14^

This case report illustrates the repair of a displaced, comminuted fracture of the right zygomatic process, using a 2.7‐mm LCP, which to the authors’ knowledge, has not been reported before. LCPs have been used successfully for a variety of fractures^15^ and allow complete load bearing across the fracture gap without relying on bone‐to‐plate friction for stability, subsequently able to withstand higher loads than other plates. While high loads are unlikely in this area, the LCP has another advantage of preserving periosteal perfusion. The LCP's thin construct (3.5‐mm) was ideal in this location where skin maneuverability is limited and resulted in lower tension on the incision. Use of the LCP resulted in complete fracture healing and excellent cosmesis (Figure 3).

Disruption of motor innervation to the upper eyelid was an unfortunate complication. We are unable to say whether this was present at the time of admission or due to placement of the LCP, as soft tissue swelling precluded a thorough assessment of function on presentation, and obscured surgical vision of the pathway of the auriculopalpebral nerve. We initially believed that there was bruising and inflammation to the nerve branch (Type 1 neuropraxia). This type of injury is transient and usually resolves within 3‐6 weeks.^16^, ^17^ However, following plate removal, and the complete loss of palpebral response, an axonometric injury was presumed. In human literature, spontaneous reversal of a peripheral nerve can take anywhere from a couple of days to weeks depending on the type of damage and location.^18^ To the authors’ knowledge, there are no reports detailing resolution of these injuries in horses. Resolution periods have been extrapolated from injuries such as “Sweeney,” where a minimum of 70 days is required before some improvement is noted, with axonal sprouts growing at 1 mm/d,^16^, ^19^ based on the specialist veterinary ophthalmologist experience managing this case externally (N. Mitchell, personal communication), permanent tarsorrhaphies are currently indicated if no response is observed within 4‐6 weeks. Our case suggests that a longer period of time should be given to such cases, especially if cosmesis is a significant factor in the outcome.

The development of contralateral limb juvenile osteoarthritis (OA) of the proximal interphalangeal joint was unfortunate, and the specific etiology was unclear.^20^, ^21^

## 7 CONCLUSION

This case report demonstrates the excellent cosmetic outcome and healing of a depressed, comminuted fracture of the zygomatic process in a 4‐month‐old foal using an LCP, which has not been previously reported on.

We believe this report suggests that intensive medical management and a prolonged rehabilitation period can be beneficial in cases of periorbital trauma when cosmetic outcome is a significant factor.

## CONFLICT OF INTEREST

None declared.

## AUTHOR CONTRIBUTION

All authors were involved in the diagnosis, surgical treatment, and postoperative management of this case. All authors contributed equally to the case report design and layout and were involved in the revision process.
